# Structural basis of signaling of cannabinoids receptors: paving a way for rational drug design in controling mutiple neurological and immune diseases

**DOI:** 10.1038/s41392-020-00240-5

**Published:** 2020-07-21

**Authors:** Shenming Huang, Peng Xiao, Jinpeng Sun

**Affiliations:** 1 Department of Biochemistry and Molecular Biology, School of Basic Medical Sciences, Key Laboratory Experimental Teratology of the Ministry of Education, Cheeloo college of Medicine, Shandong University, 250012 Jinan, Shandong China; 2 Department of Physiology and Pathophysiology, School of Basic Medical Sciences, Key Laboratory of Molecular Cardiovascular Science of the Ministry of Education, Peking University, 100191 Beijing, China

**Keywords:** Structural biology, Structural biology

Cannabinoids (CBs), analgesic drugs used for thousands of years, were first found in *Cannabis sativa*, and the multiple CBs used medicinally, such as tetrahydrocannabinol (THC), cannabidiol (CBD) and dozens more, have complex structures. In addition to their production by plants, CBs are naturally present in the nerves and immune systems of humans and animals. Both exogenous and endogenous CBs carry out a variety of physiological functions by engaging with two CB receptors, the CB1 and CB2 receptors, in the human endocannabinoid system (ECS). Both CB1 and CB2 are G protein-coupled receptors that share a 7-transmembrane (7TM) topology. CB1, known as the central CB receptor, is mainly distributed in the brain, spinal cord, and peripheral nervous system. CB1 activation in the human body typically promotes the release of neurotransmitters, controls pain and memory learning, and regulates metabolism and the cardiovascular system. Clinically, CB1 is a direct drug target for drug addiction, neurodegenerative diseases, pain, epilepsy, and obesity. Unlike the exclusive expression of CB1 in the nervous system, CB2 is mainly distributed in peripheral immune cells. Selective CB2 agonists would have therapeutic potential in the treatment of inflammation and pain and avoid side effects caused by currently used clinical drugs. Although significant progress has been made in developing agonists toward CB receptors, efficient clinical drugs targeting CB receptors remain lacking due to their complex signaling mechanisms. The recent structural elucidation of CB receptors has greatly aided our understanding of the activation and signal transduction mechanisms of CB receptors.

Structural characterization of CB receptors at the atomic level began in 2016, when Professor Zhi-jie Liu’s laboratory and Dr. Zhenhua Shao in the Rosenbuam laboratory solved the crystal structure of CB1.^1,2^ This structural information greatly facilitated the understanding of CB1 ligand recognition and signal transduction mechanisms. Continuing this progress, Professor Zhi-jie Liu’s laboratory determined two agonist-bound CB1 crystal structures, which not only uncover the agonist-CB1 interactions within the orthosteric ligand-binding pocket but also disclose the different structural features of agonist-bound and antagonist-bound CB1. In 2019, Brian Kobilka’s group and Skiniotis’s group reported the cryo-electron microscopy structure of CB1 bound to an agonist, FUB, and downstream heterotrimeric Gi protein. The agonist, FUB, exhibited a high affinity for the orthostatic ligand-binding pocket of the CB1 receptor, maintaining the CB1 receptor in an active configuration to form a stable complex with nucleotide-free heterotrimeric Gi protein. The highly conserved orthosteric binding pocket of CB1 poses a great challenge for the rational drug design of potent CB1 agonists with high selectivity. Therefore, avoiding the orthosteric site and developing allosteric regulators of CB1 have become CB1 research hotspots. To address this issue, a collaborative effort by the teams of Dr. Shao Zhenhua and Dr. Rosenbuam resulted in solution of the crystal structure of CB1 in complex with an allosteric ligand.^3^ In this structure, the allosteric modulator ORG27569 was identified at the outside of the 7TM bundle of the receptor, buried in the cell membrane. This new discovery undoubtedly provides a new route for drug development toward the CB1 receptor.

Along with progress made in CB1 research, the study of another CB receptor, CB2, has also achieved great breakthroughs. In 2019, Professor Zhi-jie Liu’s laboratory solved the crystal structure of CB2 in complex with a rationally designed antagonist AM10257. This structure reveals the distinct antagonist-binding mode in CB2 and provides the molecular basis for the high-degree subtype selectivity of antagonists between CB1 and CB2. In January 2020, due to their combined efforts, the groups of Huaqiang Xu, Xiangquan Xie, and Cheng Zhang published the three-dimensional structure of CB2 bound to the agonist WIN 55212-2 and heterotrimeric Gi protein,^4^ revealing the mechanisms by which the specific agonist WIN 55212-2 activates CB2 and CB2 interacts with the Gi protein. In the same issue of Cell, Zhi-jie Liu’s group reported a systematic study on the structures of both CB1 and CB2 engaged with G proteins,^5^ and the crystal structure of agonist-bound CB2. By simultaneously solving the three-dimensional structures of the AM12033-CB2-Gi and AM841-CB1-Gi complexes, they revealed the structural basis for the activation of CB1 and CB2, as well as their coupling to downstream G proteins.

**Fig. 1:**
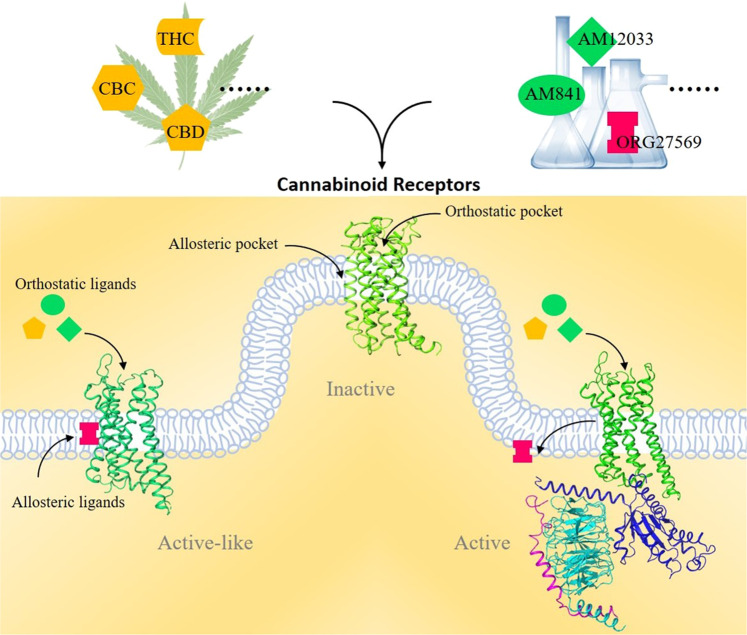
Structural understanding of Cannabinoid receptors. Recent structural studies have revealed that two cannabinoid receptors (CB1 and CB2) shared a conserved orthostatic binding pocket for their agonists. Notably, an extra allosteric binding pocket was found for CB1 receptor. Both endogenous molecule cholesterol or synthetic ligand ORG27569 was able to bind to allosteric pockets, thus regulate activation state of CB1. Collectively, crystallographic and Cryo-EM studies have identified at least three structural states for CB receptors, which are inactive, active-like (intermediate) and active, indicating complex mechanisms underlying CB receptors’ activation and signaling transduction.

CB1 and CB2 share 44% sequence homology and are simultaneously activated by many natural CB molecules. Consistently, recent structures of CB receptors have provided the structural basis for this phenomenon and shown that these two receptors share very similar ligand-binding pockets at orthosteric sites, generating great challenges in the design of selective agonists. However, structural identification of the allosteric ligand-binding sites in CB receptors provides new hope for the development of small compounds to selectively modulate CB receptor functions. These recent structural studies suggest that CB receptors adopt at least three states, an antagonistic state (inactive), intermediate state (active-like) and active state, which serves as the structural basis for complex signaling downstream of CB receptors (Fig. 1). Recent structural characterizations of CB receptors will greatly facilitate the design of new ligands to modulate the selective functions of CB receptors. Notably, the CBD was approved by the Food and Drug Administration (FDA) in 2018 to treat epilepsy. We now look forward to more drugs targeting these two CB receptors for clinical usage in the near future.
